# Lung transplant: A clinical overview

**DOI:** 10.1016/j.clinme.2025.100328

**Published:** 2025-05-15

**Authors:** Usman Feroze Khatana, Jasvir Singh Parmar, Caroline M. Patterson

**Affiliations:** a Specialty Registrar, Respiratory Medicine, Bedford Hospital, Bedfordshire Hospitals NHS Foundation Trust, Bedford, United Kingdom; b Consultant in Respiratory and Transplant Medicine, Royal Papworth Hospital NHS trust, Cambridge, United Kingdom

**Keywords:** Lung transplant, Immunosuppression, Chronic lung allograft dysfunction (CLAD)

## Abstract

Lung transplant is a recognised treatment option for end-stage respiratory failure. It is, however, a complex process and involves careful patient selection, comprehensive work-up followed by a technically challenging surgical procedure and a rigorous follow-up. In this article we provide an overview of the process, condensed down to the important facts for physicians with limited experience with transplant patients or medicine. Our aim is to take the reader through the journey from patient selection to post-transplant care and attempt to address commonly faced issues with such a niche cohort of patients.

## Highlights

• Referral for lung transplantation should be considered early in the disease trajectory.
• Appropriate patient selection is paramount for a successful outcome from lung transplantation.
• Post-transplant care is a nuanced field, and advice should be sought before carrying out any changes to the recipient’s management.
• Decisions regarding immunosuppression must be taken in consultation with the transplant team.
• Chronic lung allograft dysfunction (CLAD) is a major cause of mortality post-transplant.

## Introduction

Lung transplantation is a recognised treatment for carefully selected patients with advanced respiratory failure and can offer both prognostic and quality of life benefits. Since the first successful lung transplant, performed by Dr James Hardy and colleagues at the University of Mississippi in 1963,^1^ there have been incremental advances in patient and donor selection, organ retrieval and preservation, surgical techniques, perioperative management and immunosuppression strategies. As a result, an increasing number of lung transplant recipients are surviving and thriving in the community.

The aim of this paper is to provide an overview of the referral and listing pathway in the UK, relevant to the referring clinicians, and to provide a summary of key postoperative factors relevant to the general medical physician reviewing these patients.

## Statistics and outcomes for lung transplantation in the UK

According to the NHS Blood and Transplant (NHSBT) Annual Report on Lung Transplantation, 1,478 adult lung transplants were performed in the UK between 2014 and 2024, with 135 in the fiscal year 2023–2024.^2^

Of the 201 adult registrations onto the UK lung or heart–lung transplant waiting list in 2023–2024, 67% were male, with a median age of 56 years. The most common indication was fibrotic lung disease.^2^

Patients active on the lung transplant waiting list are stratified by urgency status based on pre-defined clinical criteria. On 31 March 2024, there were 262 adult patients awaiting lung transplant: 257 on the non-urgent list, five on the urgent list and none on the super-urgent list.^2^

The non-urgent waiting time is 530 days vs 22 days for urgent listed adult patients.^2^ There is variation in waiting time based on candidate size, primary disease group, blood group, and between the lung transplant centres.^3^

Limited donor organ availability and poor donor utilisation rates contribute to waiting list mortality. Of the 469 lungs offered from DBD (donation after brain Death) donors, 23% were transplanted and of the 386 lungs offered from DCD (donation after circulatory death) Donors, only 8.3% were transplanted.^4^ Over the same period, 41 individuals died while on the lung transplant waiting list.

Survival varies with donor and recipient characteristics and from centre to centre; however, the latest reported rates of survival in the UK are 80.7% at 1 year and 56.2% at 5 years.^2^

## Selecting the right patients for referral

Transplant referral should be considered for patients with irreversible lung pathology with the physiological and psychosocial resilience to go through the process itself, for whom all other therapies have been exhausted.^5^ Table 1 provides an overview of the factors that may be considered barriers to lung transplant candidacy. Some of these barriers are modifiable.

**Table 1:** Absolute and relative contraindications to lung transplantation.^5^

	Absolute	Relative
Age		>65 years
BMI	>35 kg/m^2^	<18 kg/m^2^ or 30–35 kg/m^2^
Malignancy	Malignancy with high risk of recurrence or death	Prior malignancy: need to rule out residual or metastatic dx
Diabetes mellitus		Poorly controlled diabetes, evidence of microvascular complications
Renal dysfunction	• Acute renal failure on dialysis and low likelihood of recovery • eGFR <40 mL/min/1.73m^2^ unless being considered for multi-organ transplant	Any degree of renal dysfunction should prompt investigation and consideration of renal referral
Liver dysfunction	• Acute liver failure • Liver cirrhosis with portal HTN or synthetic dysfunction (unless being considered for multi-organ transplant)	Any evidence of liver dysfunction (biochemical or imaging) should prompt further investigation and consideration of hepatology review
Cardiovascular disease	Acute coronary syndrome within 30 days	• Left ventricular ejection fraction (LVEF) <40% (high risk), 40–50% (risk factor). • Coronary artery disease (severe CAD needs to be revascularised prior to transplant) • Prior CABG
Cerebrovascular disease	Stroke within 30 days Progressive cognitive decline	Previous stroke: individual assessment
Connective tissue disease (CTD)		Presence of CTDs (scleroderma, lupus, inflammatory myopathy)
Infection	• Active extrapulmonary or disseminated infection • Active infection with *Mycobacterium tuberculosis, Mycobacterium abcessus, Burkholderia cenocepacia* • HIV with detectable viral load • Septic shock	• Chronic or resolved infections such as hepatitis B/C and HIV may be considered if viral load is undetectable with treatment and no evidence of other organ damage • Extensive lung cavitation with evidence of aspergillus
Haematological	Untreatable haematological disorders including bleeding diathesis, thrombophilia or severe bone marrow dysfunction	Thrombocytopenia, leukopenia or anaemia with high likelihood of persistence after transplant
Chest wall abnormalities	Significant chest wall/spinal deformity impeding adequate surgical access	• Chest wall or spinal deformity expected to cause restriction after transplant • Mediastinal fibrosis • Unusual vascular anatomy • Previous thoracic surgery or pleural intervention • Extensive pleural disease
Functional status	Limited functional status with poor rehabilitation potential	Frailty
Substance use or dependence	Active substance use or dependence (tobacco, vaping, marijuana, IV drug use)	
Psychosocial	Repeated episodes of non-adherence without evidence of improvement	Psychiatric, psychological or cognitive conditions with potential to interfere with medical adherence without sufficient support systems

## Referral

There are five adult and two paediatric lung transplant centres in the UK and referral is most commonly via a standardised proforma.

The primary indications for lung transplant referral in adults are fibrotic lung disease, COPD/emphysema, suppurative lung disease (including bronchiectasis and cystic fibrosis) and pulmonary hypertension. Referrals for heart–lung transplantation are most commonly related to congenital heart disease.

The International Society for Heart and Lung Transplantation (ISHLT) has produced a consensus document proposing criteria for transplant referral across disease groups, referenced in Table 2.^5^ However, clinicians should assess the trajectory of the underlying disease process and aim to refer earlier rather than later to facilitate candidate evaluation, optimisation and education.

**Table 2:** Criterion to trigger a lung transplant referral.^5^

Disease	Criterion for referral
Chronic obstructive pulmonary disease	BODE score 5–6 with additional factor(s) present suggestive of an increased risk of mortality: • Frequent acute exacerbations • Increase in BODE score >1 over past 24 months • Pulmonary artery to aorta diameter >1 on CT scan • FEV_1_ 20–25% predicted Clinical deterioration despite maximal treatment. Poor quality of life unacceptable to patient
Interstitial lung disease	Referral should be made at the time of diagnosis, even if a patient is being initiated on therapy, for histopathological UIP or radiographic evidence of a probable or definite UIP pattern. Any form of pulmonary fibrosis with FVC of <80% predicted or DLCO <40% predicted. Any form of pulmonary fibrosis with one of the following in the past 2 years: • Relative decline in FVC ≥10% • Relative decline in DLCO ≥15% • Relative decline in FVC ≥5% in combination with worsening of respiratory symptoms or radiographic progression Supplemental oxygen requirement either at rest or on exertion, For inflammatory ILDs, progression of disease despite treatment. For connective tissue disease ILD or familial pulmonary fibrosis, early referral is recommended as extrapulmonary manifestations may require special considerations.
Cystic fibrosis	Referral for lung transplantation should occur for a patient with CF meeting any of the following criteria despite optimal medical management, including a trial of elexacaftor/tezacaftor/ivacaftor if eligible: • FEV_1_ <30% predicted in adults • FEV_1_ <40% predicted in adults and anyone of the following: ○ 6MWT distance <400 m ○ pCO_2_ >6.6 ○ Hypoxaemia at rest or with exertion ○ Pulmonary hypertension (PA systolic pressures >50 mmHg on echo or evidence of right ventricular dysfunction ○ Worsening nutritional status despite supplementation ○ Two exacerbations per year requiring intravenous antibiotics ○ Massive haemoptysis (>240 mL) requiring bronchial artery embolisation ○ Pneumothorax • FEV_1_ <50% predicted and rapidly declining based on lung function testing or progressive symptoms • Any exacerbation requiring positive pressure ventilation
Pulmonary arterial hypertension	ESC/ERS intermediate or high risk or REVEAL risk score ≥8 despite appropriate PAH therapy Significant RV dysfunction despite appropriate PAH therapy Need for IV or SC prostacyclin therapy Progressive disease despite appropriate therapy or recent hospitalisation for worsening of PAH Known or suspected high-risk variants such as PVOD/PCH, scleroderma, large and progressive pulmonary artery aneurysms Signs of secondary liver or kidney dysfunction due to PAH Potentially life-threatening complications such as recurrent haemoptysis

## Assessment

Transplant teams most often review referred patients in person. If the patient is a potential candidate for transplant listing, a comprehensive inpatient assessment is carried out including full lung function, a 6-minute walk test, diaphragmatic studies, CT chest, ECG, echocardiography, and screening for comorbidities including coronary artery disease, gastro-oesophageal reflux and indolent/chronic infection. There is specialist input from physiotherapy, psychology, palliative care nursing, medical, surgical and anaesthetic members of the MDT.

Time is dedicated to informing patients and their families about the individualised risks and the benefits of lung transplantation. Useful information is available on the NHSBT website.^6^

The inpatient assessment process concludes with a listing MDT. If the patient is deemed to be eligible to be listed, and wishes to proceed, they will be activated on the waiting list.

Patients listed for lung transplantation are generally accepted as being those for whom no other treatment option is available, who have a greater than 50% chance of death within 2 years without transplantation, and a projected postoperative survival of at least 5 years.

## Pre-transplant care

Candidates on the waiting list should be supported to participate in rehabilitation and may require nutritional support. They are encouraged to remain up-to-date with routine vaccinations, dental checks and age-related cancer screening. Palliative care is recommended as an adjunct to disease-focused care.

When patients on the lung transplant waiting list present to hospital, it is advisable to contact their transplant team. On occasion, this may result in the individual being placed on hold on the transplant list or an adjustment being made to their listing urgency status. It is especially important that patients are discussed with the transplant team before any intervention which may potentially interfere with their transplant procedure (eg pleural intervention, commencement of anticoagulation).

For a very small number of patients who experience rapidly progressive respiratory failure but remain otherwise physically robust, extracorporeal membrane oxygenation (ECMO) may have a role as a bridge to lung transplantation.

## Transplant

Donor–recipient matching involves blood group, HLA and size compatibility. The surgical procedures for lung transplantation are single lung transplant (SLT), bilateral sequential single lung transplant (BSSLT), bilateral lobar and heart–lung transplantation. BSSLT is the most commonly performed.

The surgical approaches are via clamshell incision, bilateral anterolateral thoracotomies or sternotomy. Mechanical circulatory support intra-operatively may include cardiopulmonary bypass or ECMO.

Patients are typically weaned from ventilatory support within 24–72 h and are mobilised as early as possible after surgery. Other organ systems are supported as required. The main hazards during this period are primary graft dysfunction (PGD; a form of acute lung injury), surgical complications (bleeding, anastomotic issues, airway dehiscence), infection and, to a lesser extent, acute rejection.

Standard perioperative antibiotic prophylaxis provides cover against both Gram-positive and Gram-negative organisms, including *Pseudomonas* and *Staphylococcus aureus;* examples include tazocin and vancomycin. In addition to this, antifungal cover in the form of azoles such as itraconazole and nebulised amphotericin is part of the prophylactic regime. Co-trimoxazole is continued lifelong for *Pneumocystis* prophylaxis. Viral prophylaxis is dependent on donor and recipient CMV status. Therapy is individualised in the event of colonisation with known pathogens.

The standard immunosuppression regimen after lung transplant consists of calcineurin inhibitor such as tacrolimus, steroids such as prednisolone and mycophenolate mofetil, with all three agents generally continued lifelong. Tacrolimus is now routinely used as the first-line calcineurin inhibitor over the previously preferred ciclosporin.^7^ Some centres also prescribe induction immunosuppression with a T-cell antibody preparation (eg anti-thymocyte globulin).

## Complications associated with transplant

Beyond the immediate perioperative period, the lung remains open to the atmosphere. The combination of exposure to infection, pollution and occupational irritants, along with the need for high levels of immunosuppression, creates a challenging situation.

The airway anastomosis is the most vulnerable site for operative complications. The anastomotic site is prone to bacterial and fungal infection and there is a risk of airway necrosis, impaired healing with granulation, and subsequent airway stenosis. Complete bronchial dehiscence is exceedingly rare and may follow severe PGD or pneumonia in the early postoperative period.

The risk of acute rejection is highest in the first few months after transplant and decreases over time. Chronic lung allograft dysfunction (CLAD) is the main cause of death beyond 1 year post-transplant. The primary manifestation of CLAD is the development of airflow limitation, caused by obliterative bronchiolitis; however, up to 30% of patients with CLAD demonstrate a restrictive allograft syndrome (RAS).^8^

As the duration of immunosuppression exposure increases, lung transplant recipients are at increasing risk of malignancy, chronic kidney disease and cardiovascular disease, diabetes mellitus, GORD, osteoporosis, hypertension, hyperlipidaemia and mood disturbance.^9^

## Post-transplant follow-up

Patients are offered life-long follow-up in the post-transplant clinic. Each appointment involves radiographic imaging, spirometry, immunosuppressive drug levels, routine labs, quality of life evaluation, a nursing and medical review. Some patients will also maintain follow up with their pre-transplant medical teams, especially those with cystic fibrosis.

## Lung transplant patients at the emergency department and the general practice

Outside their transplant centres, lung transplant recipients present with issues that may or may not be related to their transplanted lungs. Transplant teams are readily available to offer advice and support when needed.

It is imperative that no decision is made regarding the withholding or adjustment of immunosuppression without discussion with the transplant team. Furthermore, no new medication should be commenced without considering the potential drug interactions. Table 3 lists possible interactions of commonly used drugs with immunosuppressive agents such as tacrolimus.

**Table 3:** List of commonly occurring drug interactions.

Interaction	Interacting drug
Increase plasma level of calcineurin inhibitor	Inhibitors of CYP3A4 including: Clarithromycin, erythromycin Azole antifungal agents (eg fluconazole, itraconazole, posaconazole, voriconazole, ketoconazole) Grapefruit juice Letermovir Paxlovid
Decrease plasma level of calcineurin inhibitor	Inducers of CYP3A4 including: Phenytoin, phenobarbital, carbamazepine, rifampicin, isoniazid, ritonavir, efavirenz
Increase risk of nephrotoxicity	Aminoglycosides AmBisome® (liposomal amphotericin) Foscarnet NSAIDs (avoid) Tenofovir disoproxil fumarate (TDF)
Increase risk of hypomagnesaemia	Proton pump inhibitors
Increase risk of hyperkalaemia	ACE inhibitors Potassium-sparing diuretics

Imaging from transplant recipients may be difficult to interpret and should ideally be reported in comparison to imaging from the transplant centre with the input of an experienced thoracic radiologist.^10^^,^^11^

Transplant teams are especially reliant on general practitioners to support chronic disease management, particularly relating to hypertension, diabetes and chronic kidney disease. These should be managed in accordance with NICE guidelines.^9^ Patients should be supported to receive routine vaccinations, but^12^^,^^13^ should not receive live vaccines.^12^

## Funding

This article did not receive any specific grant from funding agencies in the public, commercial, or not-for-profit sectors.

## CRediT authorship contribution statement

**Usman Feroze Khatana:** Writing – review & editing. **Jasvir Singh Parmar:** Writing – review & editing. **Caroline M. Patterson:** Writing – review & editing.

## Declaration of competing interest

The authors declare that they have no known competing financial interests or personal relationships that could have appeared to influence the work reported in this paper.
